# Molecular Detection of Peripheral Blood Breast Cancer mRNA Transcripts as a Surrogate Biomarker for Circulating Tumor Cells

**DOI:** 10.1371/journal.pone.0074079

**Published:** 2013-09-18

**Authors:** Adriana Lasa, Arnal Garcia, Carmen Alonso, Pilar Millet, Mónica Cornet, Teresa Ramón y Cajal, Montserrat Baiget, Agusti Barnadas

**Affiliations:** 1 Genetics Department, Hospital de la Santa Creu i Sant Pau and CIBERER U705, Barcelona, Spain; 2 Medical Oncology Department, Hospital de la Santa Creu i Sant Pau, Barcelona, Spain; 3 Medicine Department, Universitat Autònoma de Barcelona, Bellaterra, Spain; Rutgers - New Jersey Medical School, United States of America

## Abstract

Circulating tumor cells (CTCs) are becoming a scientifically recognized indicator of primary tumors and/or metastasis. These cells can now be accurately detected and characterized as the result of technological advances. We analyzed the presence of CTCs in the peripheral blood of patients with metastatic breast cancer by real-time reverse-transcription PCR (RT-qPCR) using a panel of selected genes. The analysis of a single marker, without an *EpCAM* based enrichment approach, allowed the positive identification of 35% of the metastatic breast cancer patients. The analysis of five genes (*SCGB2*, *TFF1*, *TFF3*, *Muc1*, *KRT20*) performed in all the samples increased the detection to 61%. We describe a sensitive, reproducible and easy to implement approach to characterize CTC in patients with metastasic breast cancer.

## Introduction

Breast cancer is one of the most common malignant tumours and is the major cause of death in women in the developed world. Mortality for this disease has decreased in the last years due to screening mammography programs, more precise surgery, and the efficiency of new treatments [1], [2]. Nevertheless, about 20–30% of patients with node negative breast cancer relapse and die in the following years due to the development of metastases during the course of the disease.

The appearance of metastasis depends on the migratory capacity of cells (circulating tumour cells) from the primary tumor across the lymphatic or blood vessels to distant organs [3]. The presence of circulating tumor cells (CTCs) has been associated with survival and disease progression in patients with metastatic breast cancer [4], [5]. CTCs have also been observed in neoadjuvant-treated breast cancer patients and in women with suspected breast cancer [6], [7].

Recent technological advances have allowed the development of a variety of methods to accurately detect and characterize CTCs [8], [9], [10]. These methods are based on an initial enrichment step of the sample to increase sensitivity, followed by later detection of tumour cells. Most CTC enrichment methods include either density-gradient centrifugation to extract mononuclear cells, physical filtration with commercial filter pores (isolation by size of epithelial tumor cells (ISET)) [11] or immunomagnetic separation against surface molecules commonly expressed on malignant epithelial cells. The application of microfluidics-based technologies for CTC separation is an attractive alternative which facilitates automation for high throughput sample processing. Detection of CTCs may involve either the use of monoclonal antibodies specific for epithelial cells combined with image, flow or laser scanning cytometry [12], or real time reverse transcription PCR (RT-qPCR) markers for epithelial specific transcripts [13]. The CellSearch System® (Veridex LLC, Raritan, NJ) is the only FDA-approved technique which allows the detection and quantification of CTCs [14].

The number of articles describing single or multiple markers to characterize CTCs using RT-qPCR in the blood of breast cancer patients has increased greatly in recent years [15]–[19]. The markers most frequently studied are cytokeratins, mammaglobin (*SCGB2*) and *HER2*. Cytokeratin 19 (*KRT19*) is a cytoskeletal component present in normal and cancerous epithelial cells [20]. Mammaglobin is a member of a family of epithelial secretory proteins and is considered to be a specific breast marker whose expression is confined to the mammary gland [21]. *HER2* is a member of the epidermal growth factor receptor (EGFR/ErbB) family. Its amplification plays an important role in the pathogenesis and progression of certain aggressive types of breast cancer [22]. Additional markers, often associated with colon, breast, ovarian, lung and pancreatic cancers, have also been studied. These include *EpCAM* (Epithelial cell adhesion molecule), *CEACAM* (Carcinoembryonic antigen-related cell adhesion molecule), *Muc1* (Mucine 1), *KRT20* (cytokeratin 20), *Maspin*, *EGFR* (epidermal growth factor receptor), *hTERT* (human telomerase), and *EPHB4* (ephrin receptor) [15], [19], [23].

Two further markers merit inclusion in CTC studies: *TFF1* (trefoil factor 1 or pS2) and *TFF3* (trefoil factor 3 or human secretory protein p1.B). These markers belong to the family of “trefoil” proteins whose functions are not defined, but they may protect the mucosa from insult, stabilize the mucus layer, and affect healing of the epithelium. The abnormal expression of these genes in several malignancies suggests a promotional role in tumorigeneis [24].

Although much research focuses on the prognostic role of CTCs detected by RT-qPCR in breast cancer, no consensus has been established regarding the biological markers to be used to identify these cells. The heterogeneity of experiments in studies to date does not allow conclusions to be drawn on the superiority of a specific group of markers. This may be due to differences in the detection methods, in the selection of patients, in types and number of target genes, and in the studied blood fraction.

In this study we aimed to develop a specific multimarker panel for the RT-qPCR based characterization of CTC in the blood of metastatic breast cancer patients. We analyzed the role of eight genes (*SCGB2*, *TFF1*, *TFF3*, *Muc1*, *KRT20*, *KRT19*, *EpCAM*, *CEACAM*) as CTC markers in these patients.

## Materials and Methods

### Ethical Standards

The experiments described in the paper comply with the current laws of our country.

### Cell Line and Assay Validation

The BTB-474 human breast cancer cell line (ATCC) was used to ensure that all the experimental parameters resulted in a highly efficient, sensitive and reproducible experiment.

RNA was purified from BTB-474 culture cells and diluted to different concentrations: 1000, 100, 10, 1, 0.1 and 0.01 ng/µl equivalent to 10^5^, 10^4^, 10^3^, 10^2^, 10 and 1 circulating tumor cell or CTC equivalent (CTC-EQ) respectively. One µg of each RNA dilution was retrotranscribed and amplified as described in the “Reverse transcription, pre-amplification and quantitative PCR analysis section”. Quantitative PCR was performed on each dilution using at least three replicates. A standard curve was generated by plotting the dilution series of the template against the C_q_ (quantification cycle) for each dilution. The slope of the curve was used to determine the reaction efficiency.

The sensitivity of each target gene was assessed by measuring whether a given low amount of template (1CTC-EQ) fitted the standard curve while maintaining the desirable efficiency. The standard curve also included a R^2^ (correlation coefficient) value, a measure of replicate reproducibility.

### Blood Collection and Patients

Peripheral blood was collected from 41 metastatic breast cancer patients. All patients had advanced disease confirmed by histological or imagining techniques (all of them had thoracic and abdominal CT and bone scintigraphy) and were treated in the Medical Oncology Department at Hospital de la Santa Creu i Sant Pau. Thirty-five of the 41 patients had positive estrogen receptor status, 10 had HER-2 amplification, of whom 3 had also estrogen receptor positivity. The metastases were located in soft tissues in one case, in bone in 7 cases and in various locations (bone and lung or liver) in 33 cases. All patients were receiving second or third line systemic treatment, together with endocrine therapy in 13, anti-HER-2 targeted therapy combined with chemotherapy in 10 and chemotherapy alone in 18 (10 with taxanes and 8 with capecitabine). Peripheral blood from 34 healthy female volunteers and bone marrow samples from 10 hematological patients were used as negative controls. All samples were obtained after written informed consent was given. All participants provided their written informed consent to participate in this study. The study was performed in accordance with the ethical standards laid down in the declaration of Helsinki and was approved by the Hospital Santa Cruz y Sant Pau (HSCISP) Ethical Committee. Last certification obtained April 19th, 2012.

To avoid contamination with epithelial cells during the blood extraction procedure, the first 5 ml of blood were discarded and 7,5 ml were collected into EDTA-tubes.

Peripheral blood mononuclear cells (PBMC) were isolated from each blood sample by centrifugation through a Ficoll density gradient (Lymphoprep®, Nycomed, Oslo, Norway). Total RNA was extracted from PBMC and quantified by spectrometry.

### Reverse Transcription, Pre-amplification and Quantitative PCR Analysis

One µg of RNA was retrotranscribed in a total reaction volume of 20 µl containing MgCl_2_ 5 mM, 10X buffer, DTT 10 mM, dNTP’s 10 mM each, random hexamers 15 µM, RNAsin 20 U and 200 units of MuLV enzyme. Samples were incubated for 10 min at 20°C, 45 min at 42°C, and 3 min at 99°C, followed by 10 min at 4°C.

The resulting cDNA was diluted 5-fold with distilled water, and a volume of 5 µl was used in each amplification reaction. The primers and probes for the study of all the genes were purchased already made (Assay on Demand®) from Applied Biosystems.

To improve the sensitivity of the PCR, a pre-amplification reaction of 10 cycles was performed using a pooled mixture of all the PCR assays. This pre-amplification resulted in a mean improvement of 6.54±0.33 C_q_ values and revealed no differences in the pre-amplification uniformity values of all the tested assays.

PCR reactions were set up in MicroAmp® optical 96 well reaction plates in a 20 µl reaction volume using TaqMan Universal PCR Master Mix and the Assays on Demand® according to the manufacturer’s instructions. After 2 min at 50°C, the amplification was carried out by 40 cycles at 95°C for 15 s and 65°C for 60 s in the ABI PRISM 7500 Sequence Detector System (Applied Biosystems, Foster City, CA, USA.

The following assays targeting specific mRNAs were included in the study: Hs00761767 for *KRT19* (NM_002276), Hs Hs00300643 for *KRT20* (NM_019010.2), Hs00267190 for *SCGB2* (NM_002411.2), Hs00173625 for *TFF3* (NM_003226.2), Hs00907239 for *TFF1* (NM_003225.2), Hs00158980 for *EpCAM* (NM_002354.2), Hs00989786 for *CEACAM* (NM_001184813.1) and Hs00536495 for *Muc1* (NM_058173.2).

Each sample was analyzed in triplicate. Negative controls included samples without reverse transcriptase enzyme, samples where total RNA and cDNA was replaced with genomic DNA and samples where water is used instead of template.

Quantitative values were obtained from the quantification cycle (C_q_) at which the fluorescent signal reached the threshold value between 0.1 and 0.5. ΔC_T_ values were calculated by normalizing the average expression of the target gene to the average expression of the reference gene (*GAPDH*, *B2* and *HPRT1*).

The ΔΔC_T_ method was used for relative gene expression analysis, and the average ΔC_T_ of the healthy controls for each target gene was used as the calibrator sample. The amount of target, normalized to an endogenous reference and relative to a calibrator, is given by 2^–ΔΔCT^. This method assigns a value of 1 to the calibrator sample, and all other quantities are expressed as an n-fold difference relative to the calibrator.

A sample was considered positive for a target gene if its relative gene expression was between 10 and 15-fold the highest value of the control samples.

## Results

### Efficiency, Sensitivity and Reproducibility of the Assays

**Figure 1:**
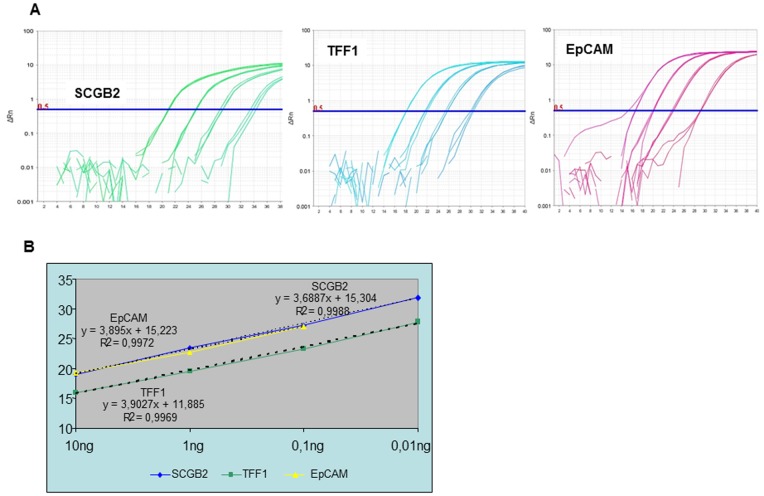
Efficiency, sensitivity and reproducibility of the assays. A) Amplification curves for a 10-fold dilution series for *SCGB2*, *TFF1* and *Muc1* gene. As the template amount decreases, the cycle number at which significant amplification is seen increase. B) Example of a standard curve and illustration of amplification efficiencies between targets. A standard curve shows the quantification cycle (Cq) on the y-axis and the starting quantify of RNA on the x-axis. Slope, y-intercept, and correlation coefficient values provide information about the performance of the reaction.

The slopes of the log phase of the amplification reaction for all the tested genes were close to -3.32. This value corresponded to efficiency close to 100%. As an example, Figure 1 shows the amplification plots and the corresponding standard curves for three of the target genes, *SCGB2*, *TFF1* and *Muc1*. The parallel nature of the blue, yellow and green curves indicated that all the amplification reactions had similar efficiencies and could therefore be accurately compared at any dilution, a requirement for the ΔΔCT calculation. A similar pattern was obtained in the case of the reference genes (*GAPDH*, *B2* and *HPRT1*).

The sensitivity of the reaction of each target gene was determined by the most diluted sample that fitted the standard curve. For the 1CTC-EQ point dilution, no amplification was detected for any of the assays. The variation in C_q_ values in the 10 CTC-EQ samples between triplicates was higher than 2.3, thus not fitting the standard curve. Sensitivity was higher when a pre-amplification step of 10 cycles was added to the protocol. A good amplification curve was obtained for all tested genes in 1 CTC-EQ and the efficiency of the standard curve was close to 100%.

The correlation coefficients, also shown in Figure 1, were between 0.996 and 0.999, reflecting the linearity of the standard curve.

### RT-qPCR Analysis

Having established the feasibility of our approach, samples of peripheral blood from 41 metastatic breast cancer patients, 10 haematological tumors, and 34 healthy female volunteers were tested for the expression of the eight selected genes. For the analysis of *SCGB2*, we additionally studied samples from 9 further female volunteers.

We used the quantitative values of three reference genes (*GAPDH*, *HPRT1* and *B2M*) to normalize the expression value of our panel of candidate genes. Fourteen out of 41 (34%) blood samples from metastatic breast cancer patients showed positive *SCGB2* expression, while no amplification occurred in samples from peripheral blood of the 34 healthy female volunteers or from the bone marrow of 10 hematological patients. Due to the high specificity of these results we classified the *SCGB2* gene as an “excellent” CTC biomarker.

**Figure 2:**
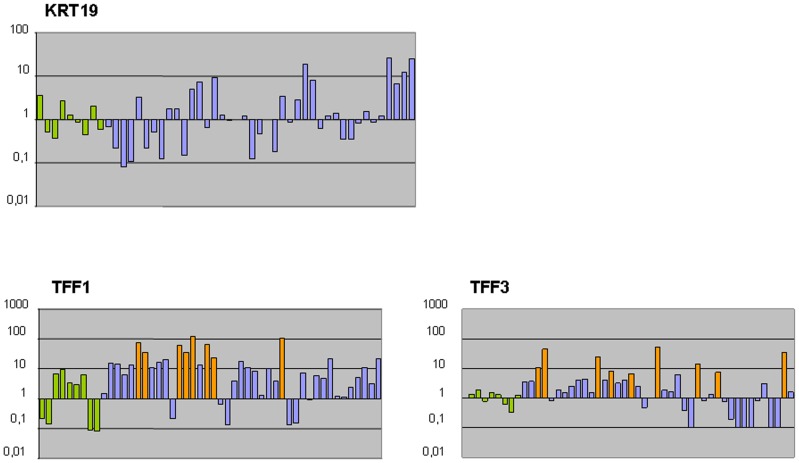
Relative expression of *KRT19*, *TFF1* and *TFF3*. Data were calculated using the ΔΔCT method, whereas the average of healthy controls was used as the calibrator sample (value = 1). Green bars indicate the values from nine healthy control samples, purple bars indicate breast cancer samples with no up regulated value and orange bars indicate breast cancer samples over expressing the target gene.

Three genes (*KRT19*, *EpCAM* and *CEACAM*) showed no specificity for breast cancer circulating tumor cells. The observed level of expression for these three biomarkers was similar in metastatic breast cancer patients and healthy volunteers (Fig. 2 and Figure S1). We considered these genes as “poor” CTC markers.

The two genes from the trefoil family (*TFF1* and *TFF3*) showed a positive expression in 7/41 (17%) and 8/41 (20%) respectively, of metastatic breast cancer patients. The expression values observed in the control group were used, as detailed at the end of the Material and Methods section, to define a positive expression value (Fig. 2). We defined these two genes as “good” CTC markers.

Finally, we considered the *Muc1* and *KRT20* genes as “fair” CTC markers considering their pattern of positive expression in 4/41 (10%) and 3/41 (7%) of metastatic breast cancer patients, respectively. There was a high heterogeneity in CTC expression between the patients. In 25 patients (61%) one or more gene expression was detected. In 16 patients were positive for 1 gene (39%), 7 patients were positive for 2 genes (17%), 1 patient was positive for 3 genes (2.4%), and 1 patient was positive for 4 genes (2.4%) (Table 1).

**Table 1:** Associations between positively expressed gene markers.

N° positivegenes	Patient ID(n = 25)	*SCGB2*+(n = 14)	*TFF3*+(n = 8)	*TFF1*+(n = 7)	*KRT19*+(n = 1)	*KRT20*+(n = 3)	*Muc1*+(n = 4)
1	CM-1	+					
1	CM-5	+					
1	CM-8	+					
1	CM-9	+					
1	CM-18	+					
1	CM-20	+					
1	CM-24	+					
1	CM-26	+					
1	CM-32	+					
1	CM-3		+				
1	CM-21		+				
1	CM-27		+				
1	CM-13			+			
1	CM-16			+			
1	CM-2					+	
1	CM-25						+
2	CM-4	+	+				
2	CM-30	+	+				
2	CM-6	+		+			
2	CM-7	+		+			
2	CM-40					+	+
2	CM-41					+	+
2	CM-14		+	+			
3	CM-17		+	+			+
4	CM-12	+	+	+	+		

Number of samples with positively and negatively expressed *SCGB2* and its association with the other markers. Sixteen patients were positive for 1 of the genes: 9 for *SCGB2*, 3 for *TFF3*, 2 for *TFF1*, 1 for *KRT20* and 1 for *Muc1*. Seven patients were positive for 2 genes: 2 for *SCGB2*+*TF3*, 2 for *SCGB2*+*TFF1*, 2 for *KRT20*+*Muc1* and 1 for *TFF1*+*TFF3*. One patient was positive for 3 genes: *TFF1*+ *TFF3*+*Muc1* and one patient was positive for four genes: *SCGB2*+*TFF1*+*TFF3*+*KRT19*.

## Discussion

Circulating tumor cells are considered as a promising diagnostic tool in oncology, and much effort is being put into finding sensitive and specific analytical methods for their detection and molecular characterization. Several techniques have been explored in breast cancer to identify CTCs, but in this paper we discuss only the results obtained using molecular biomarkers.

We analyzed the role of eight genes *SCGB2*, *TFF1*, *TFF3*, *Muc1*, *KRT20*, *KRT19*, *EpCAM*, and *CEACAM* as CTC markers in a series of metastatic breast cancer patients. We developed a new panel of five genes *SCGB2*, *TFF1*, *TFF3*, *Muc1* and *KRT20* that identified CTCs in the peripheral blood in 61% of patients, without an *EpCAM* based enrichment approach. We used a robust quantitative PCR method applying cut-off threshold values to compensate for the low-level illegitimate mRNA expression in hematopoietic cells [25].

Controversy concerning which molecular markers should be tested to identify CTCs in breast cancer patients has been a topic of discussion for many years. In an early series of 133 patients with invasive breast cancer, Grünewald et al [26], found that mammaglobin (*SCGB2*) transcripts were a specific marker for hematogenous spread of breast cancer cells, but that *KRT19* mRNA expression was not specific because it was expressed in 39% of healthy volunteers. In another series, however, Kahn et al [27], found *KRT19* expression to be specific for patients with invasive breast cancer. Since then, other studies have reported a significant association between *KRT19* and mammaglobin transcript level in CTCs from metastatic breast cancer patients [6], [16]. In contrast, we did not observe any correlation between the expressions of these two genes. Using an experimental design that did not include an enrichment step, we found that the level of *KRT19* expression in patients was similar to that in the control group. This lack of specificity may be explained by the fact that *KRT19* was expressed in the lymphocyte population in normal PBMC. Immunomagnetic selection for epithelial cells reduced the background *KRT19* signal to a frequency of <5% in normal donors [28]. However, it has been demonstrated that during the enrichment procedure normal-like breast cancer cells characterized by an aggressive behavior are lost. Taking this limitation into account we analyzed the non-enriched peripheral blood mononuclear cells.

Since 1999, when Zach reported that mammaglobin was a specific tool to detect CTCs in peripheral blood [29], many papers have confirmed the diagnostic and prognostic value of this gene as a specific molecular marker in breast cancer [15], [16]. We detected mammaglobin expression in 34% of the metastatic breast cancer patients while was absent in control samples (healthy volunteers and patients with hematological disorders). Due to the high specificity of our results using *SCGB2* we classified this gene as an “excellent” CTC biomarker.

The *TFF1* and *TFF3* genes have been poorly studied regarding detection of circulating tumor cells in cancer patients. Molloy et al [15], designed a multimarker platform for CTC detection in patients with early-stage breast cancer. It includes the human secretory protein *TFF3*. They found a positive QDA score in 20% of their patients, 31% of whom showed *EGP* and *TFF3* expression. In our study, *TFF1* and *TFF3* were positive in 20% and 17% of patients, respectively. When assessed in addition to *SCGB2*, CTC detection improved from 34% to 51%. We thus classified *TFF1* and *TFF3* as “good” CTC markers.

The *Muc1* gene is one of the markers included in a commercial test (AdnaTest BreastCancer®) which combines immunomagnetic tumor cell selection targeting *EpCAM* and *MUC1* followed by multiplex RT-qPCR for the transcripts *EpCAM*, *MUC1* and HER2. The overall detection rate for CTC using this test ranges from 22 to 69% [30], [31]. In our study, the analysis of *Muc1* expression was positive in 10% of patients and was considered a “fair” marker. *KRT20* marker was also included in this category as it was positively expressed in 7% of the patients.

We describe a sensitive, reproducible, low cost, and easy to implement RT-qPCR assay of five markers (*SCGB2*, *TFF1*, *TFF3*, *Muc1, KRT20*) that allows the characterization of CTCs in 61% of patients with metastatic breast cancer.

Numerous studies have focused on the prognostic role of CTCs detected by RT-qPCR but no consensus has been established regarding the biological markers to be used to identify those cells. The heterogeneity in the design of the “experiments” (selection of patients, detection methods, number of target genes, and studied blood fraction) can explain why those studies describe panels of markers which only rarely have genes in common. To solve this problem a multicenter study that standardizes protocols for the isolation and molecular identification of CTCs is needed.

Although the role of CTCs in clinical practice is not yet clear, their enumeration/profiling could serve as a real time biopsy with strong implications in the clinical management of breast cancer patients. The molecular profile of these cells may provide important information to identify therapeutic and resistance mechanisms in these cells, and add new insight into the biology of metastasis.

## Supporting Information

Figure S1 **Relative expression of** ***EpCAM*** **and** ***CEACAM*** **.** Data were calculated using the ΔΔCT method, whereas the average of healthy controls was used as the calibrator sample (value = 1). Green bars indicate the values from nine healthy control samples, purple bars indicate breast cancer samples with no up regulated value.
(TIF)
