# Supplementary figures and images for: Molecular Detection of Peripheral Blood Breast Cancer mRNA Transcripts as a Surrogate Biomarker for Circulating Tumor Cells
Source: PLoS One. 2013 Sep 18;8(9):e74079. doi: 10.1371/journal.pone.0074079 (PMC3776801; doi:10.1371/journal.pone.0074079)

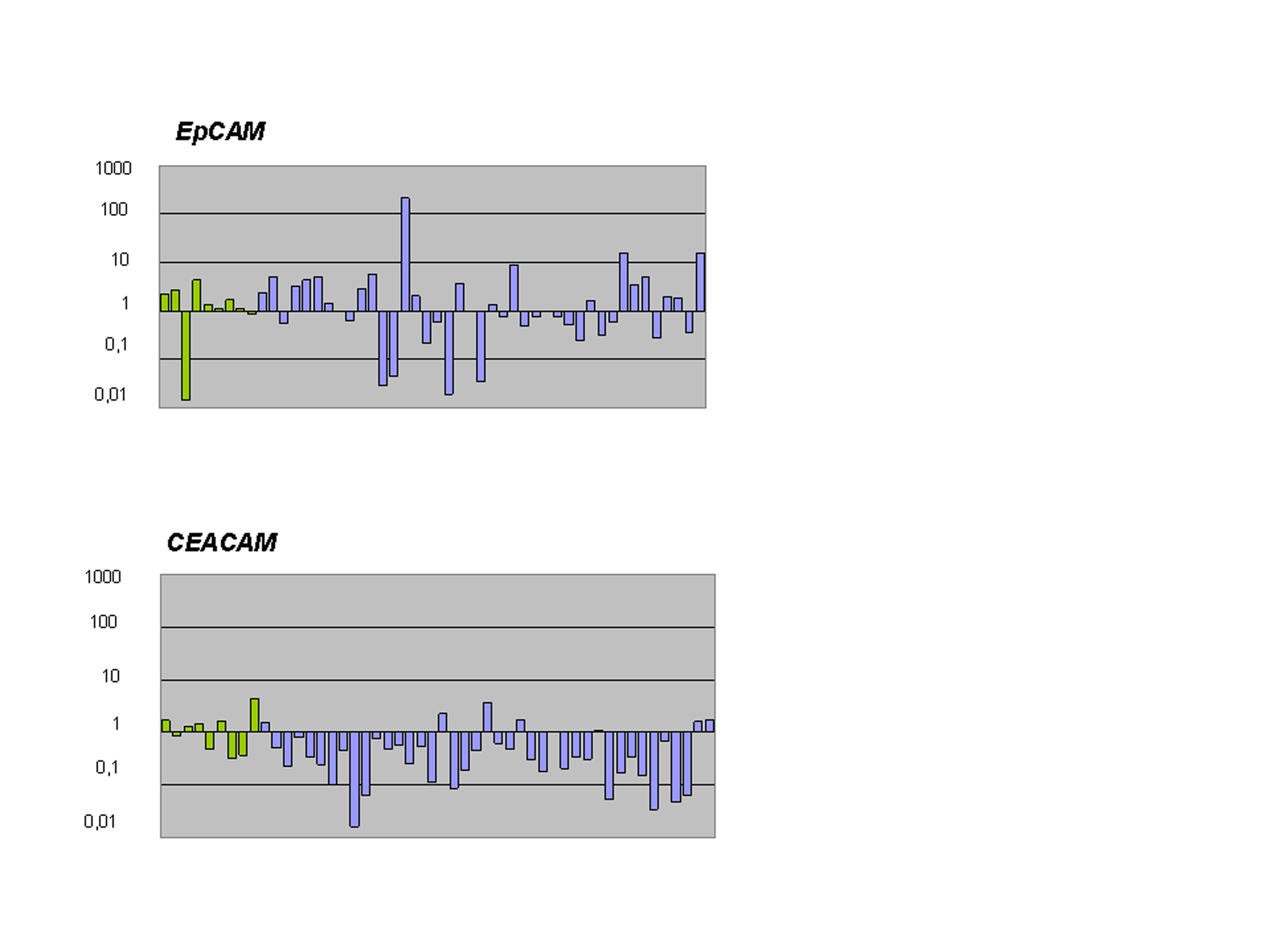

Supplement: Figure S1 — Relative expression of EpCAM and CEACAM . Data were calculated using the ΔΔCT method, whereas the average of healthy controls was used as the calibrator sample (value = 1). Green bars indicate the values from nine healthy control samples, purple bars indicate breast cancer samples with no up regulated value. (TIF) [file pone.0074079.s001.tif]
